# Experimental demonstration of a quantum router

**DOI:** 10.1038/srep12452

**Published:** 2015-07-22

**Authors:** X. X. Yuan, J.-J. Ma, P.-Y. Hou, X.-Y. Chang, C. Zu, L.-M. Duan

**Affiliations:** 1 Center for Quantum Information, IIIS, Tsinghua University, Beijing 100084, PR China; 2 Department of Physics, University of Michigan, Ann Arbor, Michigan 48109, USA

## Abstract

The router is a key element for a network. We describe a scheme to realize genuine quantum routing of single-photon pulses based on cascading of conditional quantum gates in a Mach-Zehnder interferometer and report a proof-of-principle experiment for its demonstration using linear optics quantum gates. The polarization of the control photon routes in a coherent way the path of the signal photon while preserving the qubit state of the signal photon represented by its polarization. We demonstrate quantum nature of this router by showing entanglement generated between the initially unentangled control and signal photons, and confirm that the qubit state of the signal photon is well preserved by the router through quantum process tomography.

Quantum network has many potential applications^1^,^2^. A key element to build a network is the router, which uses a control bit to determine the path of the signal bit. In a quantum router, both the control and the signal bits are represented by quantum bits in general in superposition states, and the control bits should have the ability to control the paths of the signal bits in a quantum coherent way^3^,^4^. Such quantum coherent routing of signal bits offer new remarkable opportunities compared with its classical counterpart^3^,^4^. For instance, the quantum routing operation provides the key element to realize the quantum random access memory^5^, an essential component for large scale quantum computation based on the von Neumann architecture and quantum machine learning that deals with large sets of data^6^,^7^.

In a quantum network, the signal is usually carried by single-photon pulses, which are ideal realization of the flying qubits for long-distance communication^1^,^2^. Several experiments have demonstrated routing of single-photon pulses when the control bit takes only classical states^8^,^9^. For instance, an optical switch can efficiently route single-photon pulses based on micro-electromechanical or optical control^8^,^9^. In a cavity QED (quantum electrodynamic) system, single trapped atoms or superconducting circuits are able to route the path of single photons^10^,^11^,^12^,^13^,^14^. In a genuine quantum router, the control bit may take quantum superposition states to route the paths of the signal photon in a coherent way. At the same time, the signal photon, apart from its path, need to have another degree of freedom to carry its qubit state (quantum data), which should be preserved by the quantum routing operation^4^. Such a device acts like a quantum transistor, performing entangling gates on the paths of the single-photon pulses while preserving their qubit states^1^,^2^. The ability to coherently route the path of the signal bits is critical for realization of the quantum random access memory^5^. So far no experiments have demonstrated full quantum nature of a router. The cavity QED system in principle can be used to realize a genuine quantum router^5^. However, this requires precise coherent control of both the matter and the photonic qubits, which is experimentally challenging despite the recent remarkable advance^12^,^13^,^14^.

In this paper, we report a proof-of-principle demonstration of genuine quantum routing of single-photon pulses, where the control signal, represented by the polarization of a single photon, can take arbitrary superposition states. We demonstrate the key features of a quantum router^3^: (1) the control and the signal photons take independent input quantum states, and the polarization of the control photon routes in a coherent way the path of the signal photon, generating polarization-path entanglement between the initially unentangled control and signal photons; (2) The qubit state of the signal photon represented by its polarization is well preserved by the router, so the routing operation does not destroy quantum data carried by the signal pulse. We first describe a general scheme that can realize deterministic quantum routing based on cascading of two quantum CNOT gates in a Mach-Zehnder interferometer. Deterministic quantum CNOT gates between single-photon pulses require large nonlinearity at the single-photon level, which remains experimentally daunting. Instead, in our specific demonstration, we use post-selected quantum gates based on linear optical elements^15^ and realize quantum routing in a probabilistic fashion based on post-selection by the coincidence measurements^15^,^16^,^17^. We unambiguously confirm the characteristic quantum features of the router through quantum state and process tomography. Cascading of post-selected linear optics gates has been reported before for realization of the 3-bit Toffoli gate^18^, but the scheme is different for implementation of the quantum routing operation. The implementation based on the post-selected gates can not be scaled up to many qubits. However, similar to linear optics quantum computation^19^,^20^, we can in principle make the gate and routing scheme more scalable by combining linear optics elements with feed-forward from the high-efficiency single-photon detection.

## Results

### An implementation scheme for quantum routing

**Figure 1:**
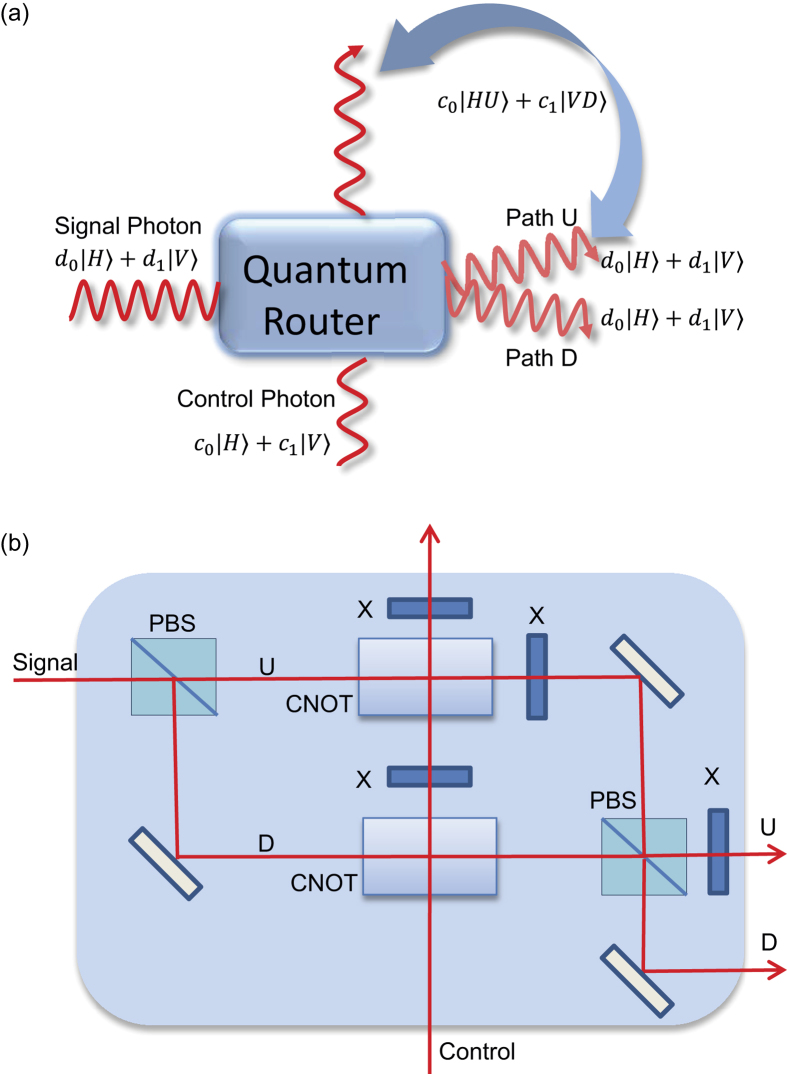
Principle and Scheme for a quantum router. (**a**) Illustration of the principle of a quantum router. A control qubit, represented by the polarization state of a single photon, routes the output paths of the signal photon in a coherent way, generating polarization-path entanglement between the initially unentangled control and signal photons. The quantum data, carried by the polarization state of the signal photon, is preserved by the routing operation. (**b**) A scheme to implement the quantum routing operation through an optical circuit with quantum CNOT gates, X gates, and an Mach-Zehnder interferometer.

The idea of a quantum router is illustrated in Fig. 1(a). The signal photon need to have two degrees of freedom: polarization and path. Its polarization is used to carry the quantum data, represented by a qubit state 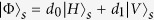 with arbitrary coefficients *d*_0_, *d*_1_, where 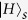 and 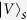 denote two orthogonal linear polarizations. The incoming path of the signal photon is denoted by 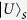. After the quantum router, the outgoing path of the signal photon is determined by an address qubit, which is represented by the polarization state of a control photon. For a classical router (optical switch), the outgoing path of the signal photon is either 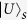 or 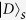, determined by the polarization of the control photon which takes either 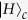 or 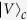. For a quantum router, the control qubit is in a quantum superposition state 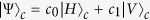 with arbitrary coefficients *c*_0_, *c*_1_, and coherence between the two classical routing possibilities should be maintained. So the routing operation generates a polarization-path entangled state 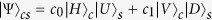 between the initially unentangled control and signal photons. Such path entanglement from quantum routing is a key requirement for realization of the quantum random access memory^5^,^6^,^7^. Similar to a classical router, which does not destroy the data carried by signal photon, a quantum router should preserve the polarization state 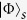 that encodes the quantum data. So, after an ideal quantum router, the final state of the system takes the form


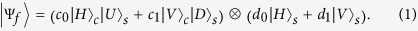


The quantum routing operation transforms the polarization and the path degrees of freedom of the signal photon in different ways, performing effectively a quantum CNOT gate on the path of the the signal photon while preserving its polarization state. This poses a challenge for the experimental realization as conditional quantum gates between the photons are typically on the polarization degrees of freedom^15^,^16^,^17^,^18^. Note that the definition of the quantum routing operation here is somewhat different from the one in Ref. 3, where after the routing the control photon and the signal photon are not in an entangled state. The polarization-path entanglement between the control and the signal photons is a key element in our approach to quantum routing as this provides the critical resource for its application in realization of quantum random access memory^5^ and in achievement of exponential speedup in large data processing^6^,^7^.

We first describe a general scheme to realize quantum routing based on cascading of two quantum CNOT gates in a Mach-Zehnder interferometer as shown in Fig. 1(b). A signal photon, initially in the polarization state 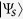 with arbitrary superposition coefficients *d*_0_, *d*_1_, is incident from one side of the polarization beam splitter (PBS) of the Mach-Zehnder interferometer. The PBS correlates the photon’s polarization and path degrees of freedom and transforms its state to 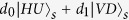. The control photon, initially in the state 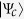, meets the signal photon successively through the paths *D* and *U*. When the two photons meet each other, we perform a quantum CNOT gate which flips the polarization of the signal photon if and only if the control photon is in *H*-polarization. The Pauli operation *X* in the circuit exchanges polarization *H* and *V* for the photon in the corresponding path. After the second PBS of the Mach-Zehnder interferometer, the output state is given by 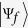 (see Supplementary Material for detailed derivation). So the optical circuit in Fig. 1(b) achieves exactly the quantum routing operation. The scheme described here is simpler and more general than the one proposed in Ref. 3: first, we don’t need the quantum non-demolition measurement required in^3^. Furthermore, this implementation scheme, by itself, is deterministic, not limited to linear optics gates, and applies to any experimental systems where one can realize quantum CNOT gates on single-photon pulses.

### Experimental setup

**Figure 2:**
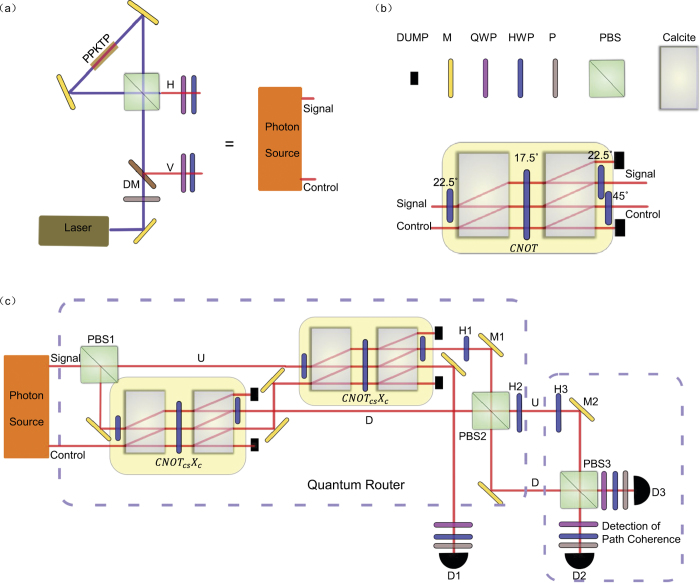
Experimental setup for realization of a quantum router. (**a**) Experimental setup for the photon source to generate control and signal photons with independently controlled arbitrary polarization states. (**b**) Experimental setup for a post-selected quantum CNOT gate between the control and the signal photons, where the numbers show the corresponding angles of the half wave plates (HWPs). (**c**) Experimental setup for the quantum router. The quantum CNOT gate *CNOT*_*cs*_, with the signal photon as the target qubit, is combined with the X-gate *X*_*c*_ on the control photon, and realized by the optical elements shown in the yellow-shaded boxes. The two big Mach-Zehnder interferometers, one by PBS1 and PBS2, and the other by PBS2 and PBS3, are both actively phase locked to maintain phase stability, where the locking laser beams and optical/electronic devices are not shown in the figure for clarity of the picture (see Methods for details). The HWPs H1 and H2 are both set at an angle of 45°, performing X gates on the photon’s polarization in the corresponding path. The optical elements in the last dash-line box is only required for detection of coherence between the output paths (*U* or *D*) of signal photon. The HWP H3 is also set at 45°. The rotation of HWPs, quarter wave plates (QWPs), and polarizers (P) before each of the single-photon detectors (D1, D2, and D3), combined together, can choose an arbitrary polarization basis for photon detection, which is required for quantum state tomography. Tomographic measurement of quantum states is performed by registering the two-photon coincidence events between the detectors D1 and either D2 or D3.

The proposed scheme in Fig. 1(b) for realization of the quantum router is deterministic if one can realize deterministic CNOT gates. As a proof-of-principle experiment, here we demonstrate a probabilistic version of this scheme using the post-selected linear optics quantum CNOT gates^15^, with the experimental setup shown in Fig. 2. This experimental setup has some similarity with the one in Ref. 21 recently exploited for implementation of quantum state fusion. However, there is an important difference: the setup in Ref. 21 does not generate polarization-path entanglement between the control and the signal photons, which is a key feature of our quantum router scheme. As first demonstrated in Ref. 15, the optical circuit shown in Fig. 2(b) realizes a post-selected CNOT gate on the two input modes, conditional on the case that one photon exits from each of the output modes, which occurs with a probability of 1/9^15^,^22^. In our experimental setup shown in Fig. 2(c), the CNOT gate is combined with the Pauli gate *X*_*c*_ on the control photon, so the last half wave plate (HWP) at 45° can be removed. The control photon after the first CNOT gate needs to go through the second CNOT gate in the upper arm of the Mach-Zehnder interferometer. The combined success probability of these two successive gates is 1/27, corresponding to the case of one photon in the control mode and the other photon in one of the interferometer arms of the signal mode (see the Supplementary Material for details on post-selection measurements). After the quantum router, we confirm quantum coherence between the two output paths of the signal photon through another Mach-Zehnder interferometer as shown in Fig. 2(c).

### Experimental results

**Figure 3:**
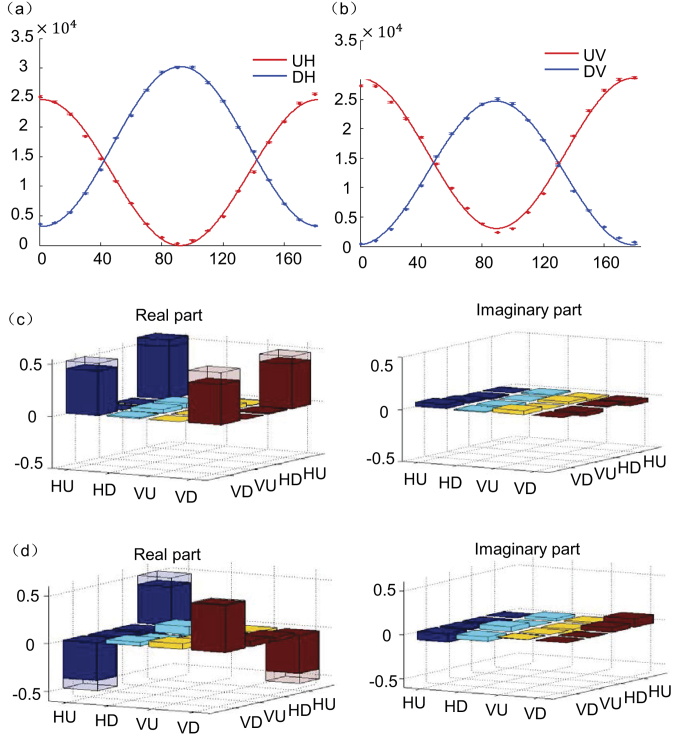
Experimental verification of a quantum router. (**a**) The detected coincidence photon counts (accumulated over 13 seconds) in different paths are shown as functions of the polarization angle *θ* of control photon. The angle *θ* varies from 0° to 180°, with the corresponding polarization state 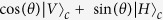. The symbols UH and DH mean that the signal photon is in U or D path with H-polarization, and the corresponding data represent the photon coincidence counts between the detectors D1 and either D2 or D3. The error bars denote standard derivation and their calculation is specified in detail in Methods. (**b**) Same as Fig. a, but with the signal photon now in *V*-polarization. (**c**) Real and imaginary parts of the reconstructed density matrix elements for the polarization-path state of control and signal photons. The input polarization state of control photon is 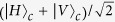 and of signal photon is 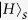. The hollow caps denote the matrix elements for the ideal output state 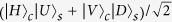 after a perfect router. (**d**) Same as Fig. c, but now the input polarization state of control photon is 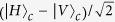 and of signal photon is 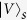.

To demonstrate quantum routing operation, first we rotate the polarization state 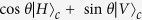 of the control photon by continuously varying *θ*, and check the output path of the signal photon prepared in *H*-polarization. The recorded coincidence counts for the signal photon in the *U* or *D* path are shown in Fig. 3(a), which follow oscillation curves cos^2^*θ* and sin^2^*θ*, respectively. The oscillation of photon counts in the *U* path has a high visibility of 97.0%, while the corresponding visibility for the *D* path is only 84.6%. The difference in visibility comes from different roles played by the CNOT gates for this scenario: as one can check from the optical circuit in Fig. 2(c), for the signal photon to go to the *D* path, the CNOT gate plays an active role to flip its polarization through two-photon Hong-Ou-Mandel interference, which typically has a larger imperfection^15^. Such a polarization flip is not required for the signal photon to go to the *U* path. The difference of these two visibilities of about 12% is therefore a characterization of the imperfection of the linear optics CNOT gate. The infidelity of the CNOT gate is mainly induced by the imperfect mode matching between different optical paths for the Hong-Ou-Mandel interference. In Fig. 3(b), we show the path information of signal photon now prepared in *V*-polarization. We observe similar oscillation curves except that the visibilities for the *U* and the *D* paths are exchanged (of 80.0% and 97.3% respectively) as the role of CNOT gate is reversed in this case.

To confirm quantum nature of this router, we demonstrate coherence between the routing paths and polarization-path entanglement generated between the control and the signal photons initially in product states. We set the input polarization state 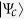 of the control photon to 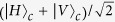. In the idea case, the output for the control and the signal photons should be in the polarization-path maximally entangled state 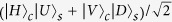. We verify this entanglement by reconstructing the density matrix for the output polarization-path state through measurements based on quantum state tomography^23^. For two-qubit states, the quantum state tomography is done with 16 independent measurements in complementary bases and the density matrix is reconstructed using the maximum likelihood method^23^. The real and imaginary parts of all the elements of density matrix are shown in Fig. 3(c) with the signal photon carrying *H*-polarization state. From this measurement, we find that the entanglement fidelity *F* of the output state is *F* = (88.5 ± 0.5)%, well above the boundary of criterion *F* > 0.5 for demonstration of entanglement^24^. The entanglement is also confirmed for other superposition states of 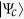 and found to be almost independent of the polarization state of signal photon. As an example, in Fig. 3(d) we show the reconstructed density matrix elements for the output polarization-path state with 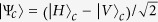 and the signal photon in *V*-polarization. The corresponding entanglement fidelity in this case is *F* = (83.0 ± 0.5)%. The small decrease in entanglement fidelity (about 5%) results from the slightly larger imperfection for one of the CNOT gates in the Mach-Zehnder interferometer, and this fidelity difference is consistent with the difference in the two visibilities (84.6% versus 80.0%) that we observed in Fig. 3(a,b).

**Figure 4:**
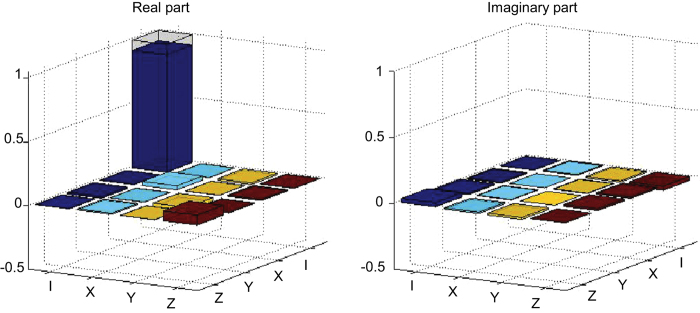
Quantum process tomography of the data qubit. Real and imaginary part of the reconstructed process matrix elements for the data qubit carried by the polarization state of signal photon. The quantum router preserves the data qubit, so in the ideal case the operation is represented by an identity operator with its elements shown by the hollow caps. The figure shows the case with the control photon in *H*-polarization. For *V*-polarization, the figure looks pretty much the same.

The quantum router should preserve quantum data carried by the polarization state of signal photon. Although the polarization of signal photon plays an important role in the Mach-Zehnder interferometer and in quantum CNOT gates, the whole quantum routing operation combining all the elements together should not change this polarization state. So, in the subspace of quantum data represented by the polarization qubit of signal photon, the router just performs an identity gate. To confirm this, we reconstruct the routing transformation in this subspace through quantum process tomography (see Methods)^25^. The reconstructed process matrix elements are shown in Fig. 4 with the control photon initially in *H*-polarization. From the result, we conclude that the process fidelity *F*_*P*_ = (91.9 ± 0.3)% and the average gate fidelity 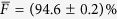. For the *V*-polarization component of control photon, the measured process matrix in the quantum data subspace is very similar and thus not shown in the figure. We find the corresponding average gate fidelity for this latter case is 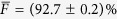.

## Discussion

Experimental demonstration of a quantum router opens up prospects for its application in quantum networks and quantum data processing. It provides the key element for realization of the quantum random access memory^5^. As the paths of signal photons get entangled with the control qubits in a quantum router, it allows us to make a delayed choice of the routing destination, either to one location or superposition of several locations, similar to the quantum delayed-choice experiments^26^. Such a delayed-choice routing, apart from its fundamental interest, may have applications in network cryptography. The control qubits perform effectively entangling gates on the paths of quantum signals. In analogy to quantum parallelism in superfast quantum algorithms, such entangling gates may be exploited for achieving constructive interference between the paths of signals, leading to quantum parallel distribution of signals in a network.

## Methods

### Derivation of quantum routing operation through the circuit in Fig. 1(b)

The input of the control and the signal photons is given by a product state |Ψ_in_〉=(c_0_|H〉_c_+c_1_|V〉_c_)⊗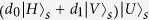 with arbitrary coefficients *c*_0_, *c*_1_, *d*_0_, *d*_1_. After the first PBS of the Mach-Zehnder interferometer, the state transforms to


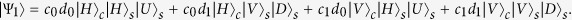


Then, after the CNOT gate at the lower interferometer arm and an *X* gate on the control photon, the state becomes


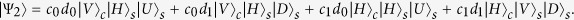


The next operation is the CNOT gate at the upper interferometer arm followed by an *X* gate on the control photon and an *X* gate on the signal photon in the *U* path. After this operation the state evolves to


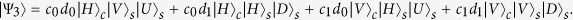


The last operation is the second PBS of the interferometer acting on the signal photon and an *X* gate on the signal photon in the *U* path. This gives the final state


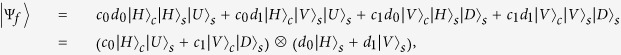


which is exactly the output state one expects from an ideal quantum router.

### Experimental details

In our experiment, the photon source is given by a pair of single photons generated through spontaneous parametric down conversion in a nonlinear periodically-poled potassium titanyl phosphate (PPKTP) crystal of 15 mm length (see Fig. 2a). The crystal is pumped by a continuous-wave (cw) diode laser at 404 nm wavelength, generating signal and control photons at 808 nm wavelength. The sagnac loop interferometer shown in Fig. 2a can generate polarization entangled photon pairs if the pump beam is set at 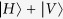 polarization. For our experiment, however, we set the pump beam at 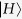 polarization by the polarizer before the dichromatic mirror (DM) so that the down-converted photons have *H* and *V* polarization, respectively, and go out along different paths after the PBS. The half wave plate (HWP) and the quarter wave plate (QWP) in the two output paths then prepare the control and the signal photons respectively to independent polarization states with arbitrary coefficients *c*_0_, *c*_1_, *d*_0_, *d*_1_.

The quantum CNOT gate in our experiment, shown in Fig. 2b, is constructed using the same method as in ref. 15. It is made of two parallel placed Calcites with a HWP between them set at an angle of 17.5^*o*^. Calcites work as a PBS, and the two parallel Calcites make a Mach-Zehnder interferometer of two loops with intrinsic phase stability. With two-photon Hong-Ou-Mandel interference in this interferometer and the HWPs set at appropriate angles given in Fig. 2b, one can check that the device performs a quantum CNOT gate on the incoming control and signal photons, provided that one post-selects the case that one photon exits from each of the two output paths^15^,^16^. The post-selection is done through photon coincidence measurement. There could be small phase difference in the optical paths of the Mach-Zehnder interferometer formed by the Calcites, which is compensated afterwards by adjusting the tilting angle of a birefringent BBO crystal (not shown in the figure). The post-selected quantum CNOT gate is subject to leakage error, where both the control and the signal photons go to one of the output paths and the final state leaks outside of the qubit Hilbert space^15^,^16^. This leakage error leads to background noise in the coincidence measurement on the outputs of the router shown in Fig. 2c. The contribution of this background noise is measured in our experiment by registering the coincidence while blocking either the control photon path or the signal photon paths after the first CNOT gate (at the lower arm of the interferometer). The background counts can then be subtracted from the total counts, which removes this noise. When all the coefficients *c*_0_, *c*_1_, *d*_0_, *d*_1_ are nonzero, interference terms may arise between the background noise and the signal terms. To remove contribution of these interference terms, we can insert a phase shifter to the path of the control photon after the first CNOT gate to induce a phase shift of either *α* (*α* can be any real number) or *α* + *π* per photon. After averaging of the coincidence counts for these two rounds of experiments with a relative phase shift of *π*, the noise interference terms are removed while the signal terms remain unaffected.

The quantum router setup shown in Fig. 2c has a big Mach-Zehnder interferometer of long arms that are subject to phase fluctuation, so the interferometer needs to be actively phase locked to maintain phase stability. A diode laser beam at 780 nm wavelength is incident from the top side of PBS1, goes through the interferometer loop, and is then separated by a dichromatic mirror after the PBS2 and detected by photon-detectors. The detected signal, after a PID circuit, provides the feedback to fine tune the position of mirror M1 in the interferometer loop through a piezo to maintain stability of the relative phase. After the router, to detect phase coherence between the two output paths of signal photon, we combine the paths through another PBS (PBS3). The path coherence is confirmed by detecting the polarization states of two outputs of the PBS3 in different bases, where the basis selection is achieved through a polarizer together with a HWP and a QWP. The detected counts by the single-photon detectors are registered through a coincidence circuit, recording a signal only when one control photon and one signal photon are detected. All the error bars in this paper result from the statistical error associated with the photon detection under the assumption of a Poissonian distribution for the photon counts. The error bars are propagated from the registered photon counts to the measured quantities (such as the density matrix elements and the entanglement fidelity) through exact numerical simulation. The PBS2 and PBS3 in Fig. 2c make another Mach-Zehnder interferometer, which requires similar phase locking, achieved through feedback to the piezo-controlled position of mirror M2 by detection of another diode laser beam going through this interferometer.

Theoretically, the success probability of the quantum routing operation shown in Fig. 2 using the post-selected quantum gates is 1/27 ≈ 0.0370. To experimentally measure this success probability, we first set the angle of the middle HWP in the CNOT optical circuit of Fig. 2(b) to 0°. At this angle, both the circuit in Fig. 2(b) and the quantum routing circuit in Fig. 2(c) reduce to a deterministic identity operation on the input photons. We record the photon coincidence counts in this case as the comparison point, which is about 6.0 × 10^4^ per second. Then we set the HWPs back to the right angle (17.5°) for the quantum routing operation and measure the coincidence counts of the output photons, which is 2.2 × 10^3^ per second. The ratio between these two coincidence counts gives the experimentally measured success probability, which is 0.0367, in agreement with the theoretical value.

For each data point shown in Figs 3 and 4, we typically collect more than 10^5^ coincidence counts in 13 seconds. The error bars are determined by assuming a Poissonian distribution for the photon counts. We propagate the error bars from the detected photon counts to the quantities shown in Figs 3 and 4 through exact numerical simulation by Monte Carlo sampling according to the Poissonian distribution of the photon counts.

### Quantum process tomography

A quantum process is described by a completely positive map *ε* which transfers arbitrary initial states *ρ*_*i*_ to the corresponding final states *ρ*_*f*_ ≡ *ε*(*ρ*_*i*_). In quantum process tomography (QPT), a fixed set of basis operators {*E*_*m*_} are chosen so that the map 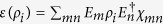 is identified with a process matrix *χ*_*mn*_. We experimentally reconstruct the process matrix *χ* through the maximum likelihood technique^25^. For the single-bit QPT, we choose the basis operators as *I* = *I*, *X* = *σ*_*x*_, *Y* = −*iσ*_*y*_, *Z* = *σ*_*z*_, which requires measurement on four different initial states |*H*〉, |*V*〉, |+〉 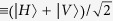, and 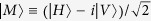. To experimentally measure the process matrix elements shown in Fig. 4, we first set the polarization of the control photon to |*H*〉 and prepare the polarization of the data photon to one of the four states 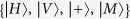. For each input state, we measure the output density matrix elements of the data photon by detecting the photon counts in three complementary bases 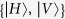, 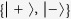, and 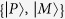, respectively, where 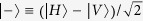 and 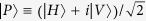. The corresponding output density matrices are determined through the maximum likelihood method as it is standard for quantum state tomography^23^. From the measured output density matrices for each of the four input states 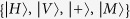, we reconstruct the experimental process matrix *χ*_*e*_ following the standard maximum likelihood method for quantum process tomography^25^, which gives the data shown in Fig. 4. By the same method, we have also measured the process matrix for the data photon when the polarization of the control photon is set to |*V*〉. Finally, we compare the experimentally reconstructed process matrix *χ*_*e*_ with the ideal one *χ*_*id*_ by calculating the process fidelity *F*_*P*_ = *Tr*(*χ*_*e*_*χ*_*id*_). The process fidelity *F*_*P*_ is connected with the average gate fidelity 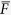 through the formula 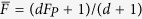
25, where 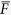 is the output state fidelity averaged over all possible input states with equal weight and *d* is the Hilbert space dimension (*d* = 2 for a single qubit).

## Additional Information

**How to cite this article**: Yuan, X. X. *et al.* Experimental demonstration of a quantum router. *Sci. Rep.* **5**, 12452; doi: 10.1038/srep12452 (2015).
